# Leveraging Controlled-Environment Agriculture to Increase Key Basil Terpenoid and Phenylpropanoid Concentrations: The Effects of Radiation Intensity and CO_2_ Concentration on Consumer Preference

**DOI:** 10.3389/fpls.2020.598519

**Published:** 2021-01-14

**Authors:** Kellie J. Walters, Roberto G. Lopez, Bridget K. Behe

**Affiliations:** Department of Horticulture, Michigan State University, East Lansing, MI, United States

**Keywords:** 1, 8 cineole, daily light integral, eugenol, linalool, methyl chavicol, *Ocimum basilicum*, sensory panel

## Abstract

Altering the radiation intensity in controlled environments can influence volatile organic compound (VOC) biosynthetic pathways, including those of terpenoids and phenylpropanoids. In turn, the concentrations of these compounds can have a profound effect on flavor and sensory attributes. Because sweet basil (*Ocimum basilicum*) is a popular culinary herb, our objectives were to (1) determine the extent radiation intensity and carbon dioxide (CO_2_) concentration influence seedling terpenoid and phenylpropanoid concentrations; (2) determine if differences in phenylpropanoid and terpenoid concentrations influence consumer preference; and (3) characterize consumer preferences to better inform production and marketing strategies. “Nufar” sweet basil was grown with CO_2_ concentrations of 500 or 1,000 μmol ⋅ mol^–1^ under sole-source radiation intensities of 100, 200, 400, or 600 μmol ⋅ m^–2^ ⋅ s^–1^ with a 16 h photoperiod to create daily light integrals of 6, 12, 23, and 35 mol *⋅* m^–2^ ⋅ d^–1^. After 2 weeks, concentrations of the terpenoids 1,8 cineole and linalool and the phenylpropanoids eugenol and methyl chavicol were quantified, and consumer sensory panel evaluations were conducted to quantify preferences. Overall, increasing radiation intensity from 100 to 600 μmol ⋅ m^–2^ ⋅ s^–1^ increased 1,8 cineole, linalool, and eugenol concentrations 2. 4-, 8. 8-, and 3.3-fold, respectively, whereas CO_2_ concentration did not influence VOCs. Contrary to our hypothesis, increased VOC concentrations were not correlated with consumer preference. However, overall liking was correlated with aftertaste and flavor. The conclusion that consumer preference is dependent on flavor can be drawn. However, increasing VOC concentrations to increase flavor did not improve flavor preference. Many consumer sensory preference characteristics (favorable preference for aftertaste, bitterness/sweetness, color, flavor, overall liking, and texture) were correlated with basil grown under a radiation intensity of 200 μmol ⋅ m^–2^ ⋅ s^–1^. This led us to determine that consumers prefer to detect the characteristic basil flavor made up of 1,8 cineole, eugenol, and linalool, which was not as prevalent in basil grown under 100 μmol ⋅ m^–2^ ⋅ s^–1^, but too high in basil grown under 400 and 600 μmol ⋅ m^–2^ ⋅ s^–1^, which led to lower consumer preference.

## Introduction

Increased demand for a year-round supply of locally grown produce in urban markets, interest in climate change resilience, and the mitigation of food deserts have spurred interest in indoor controlled-environment agriculture (CEA; Metz et al., 2007; Weber and Matthews, 2008; Reynolds and Cohen, 2016; Gomez et al., 2019; Goodman and Minner, 2019). However, the high capital and operating costs of indoor CEA can cause questionable economic feasibility of most food crops (Zeidler et al., 2013; Eaves and Eaves, 2018; Hoops et al., 2018). Therefore, the production of high-value and high-quality specialty crops such as leafy greens has prevailed in CEA, with growers indicating a need for research on manipulating the growing environment to improve crop flavor (Goodman and Minner, 2019; Walters et al., 2020).

A predominant high-value specialty crop that can vary greatly in quality is fresh cut sweet basil (*Ocimum basilicum*). Basil varies not only in visual quality, but also in flavor caused by variations in the concentration and ratios of volatile organic compounds (VOCs). Different basil species and cultivars have varying chemotypes, which can be characterized by distinct dominant compounds or ratios of compounds and therefore different overall flavors. For example, lemon basil cultivars including “Sweet Dani” contain high concentrations of citral (terpenoid), giving them a lemony flavor and aroma (Morales and Simon, 1997). Other cultivars have higher concentrations of linalool (terpenoid), methyl chavicol or eugenol (phenylpropanoids), or more than one major compound, leading to wide variation in basil flavor and aroma (Simon et al., 1999; Gang et al., 2001).

Many of the VOCs contributing to basil flavor are terpenoids or phenylpropanoids. The biosynthetic pathways for these two compound groups have differing rate-limiting steps and regulatory mechanisms. For example, terpenoid concentration is correlated with terpene synthase activity but negatively correlated with phenylpropanoid concentration and phenylalanine ammonia lyase (PAL) activity (Iijima et al., 2004). Additionally, increased energy and substrate availability generally promote VOC biosynthesis. Therefore, we hypothesized that increased concentrations of both terpenoids and phenylpropanoids could be achieved by increasing radiation intensity during production. Additionally, based on the differing biosynthetic pathway regulation, we hypothesized the ratios between compounds would differ based on the radiation intensity provided.

Based on our preliminary analysis to determine the major compounds present in sweet basil “Nufar,” two phenylpropanoids and two terpenoids were chosen for analysis (data not shown). Eugenol, a major phenylpropanoid contributing to the clove-like flavor and aroma of basil, is the major volatile oil in cloves (*Syzygium aromaticum*; Santos et al., 2009). It also has antibacterial, antifungal, and antiherbivory characteristics (Karapinar and Aktug, 1987; Sangwan et al., 1990; Moleyar and Narasimham, 1992; Sisk et al., 1996; Singh et al., 2015). The second phenylpropanoid, methyl chavicol (estragole), contributes to the anise-like aroma and flavor characteristic of basil (Simon et al., 1999). Linalool, a monoterpenoid, can be described as having an aroma and flavor of floral or spicy (Arena et al., 2006) or reminiscent of the cereal Fruit Loops^§^. Linalool also has antibacterial, antifungal, and insecticidal activity (Juliani et al., 2004). The second major monoterpenoid is 1,8 cineole (eucalyptol). With an aroma and flavor analogous to eucalyptus (*Eucalyptus globulus*), whose essential oil profile contains 70–80% 1,8 cineole (De Vincenzi et al., 2002), the compound also has insecticidal activity (Shaaya et al., 1991).

Researchers have investigated the relationship between radiation intensity and/or the daily light integral (DLI) and secondary metabolite concentrations. In general, overall VOC concentration increases as DLI increases whether the radiation is provided from the sun or artificial sole-source lighting from LEDs (Hälvä et al., 1992; Li et al., 1996; Chang et al., 2008; Kumar et al., 2013; Samuolienë et al., 2013). However, trends differ among individual compounds; this has been demonstrated in many culinary herbs including basil, dill (*Anethum graveolens*), sage (*Salvia officinalis*), and thyme (*Thymus vulgaris*) (Hälvä et al., 1992; Li et al., 1996; Chang et al., 2008; Kumar et al., 2013). In basil, linalool and eugenol concentrations increased ∼3- and 4-fold, respectively, whereas methyl eugenol decreased by ∼80%, and 1,8-cineole was unaffected as DLI increased from 5 to 25 mol ⋅ m^–2^ ⋅ d^–1^ (Chang et al., 2008). Dou et al. (2018) determined that increasing the DLI from 9 to 18 mol *⋅* m^–2^ ⋅ d^–1^ not only increased basil fresh mass, net photosynthesis, and leaf area and thickness, but also increased anthocyanin, phenolic, and flavonoid concentrations.

Marketing is the science and art of building marketing strategies to gratify consumer preferences by exploring, creating, and delivering value at a profit (Kotler and Keller, 2009). Therefore, if a production goal is to produce a premium quality product with improved flavor characteristics, connecting the concentration of VOCs that contribute to flavor with consumer preferences could improve crop flavor, consumer demand, and willingness to pay a premium. Although researchers have investigated the influence of DLI on secondary metabolite accumulation, there are limited data on how these aroma and flavor profile changes affect consumer preference. Sensory analysis panels have been conducted to determine perceived differences in basil aroma due to radiation source, radiation quality, and temperature during production (Chang et al., 2007; Seely, 2017); to determine perceived differences in drying methods (Díaz-Maroto et al., 2004; Calín-Sánchez et al., 2012); and to characterize basil cultivars (D’Antuono et al., 2007; de Costa et al., 2014; Bernhardt et al., 2015; Tangpao et al., 2018). Although some of these studies connect production practices with consumer preference, recommendations based on radiation intensity are needed.

While increasing crop quality is one method to improve economic feasibility, high-density planting is another strategy utilized to mitigate the high input costs of CEA by reducing the cost per plant. Plant density is generally greatest during the seedling production stage versus the finishing stage(s). Therefore, if greater inputs are used during the seedling stage, the cost can be spread across more plants. To leverage the environmental control capabilities of CEA to improve VOC concentrations and ratios most efficiently, our objectives were to (1) determine the extent radiation intensity influences key sweet basil seedling terpenoid and phenylpropanoid concentrations; (2) determine if differences in key phenylpropanoid and terpenoid concentrations due to radiation intensity influence consumer preference; and (3) characterize consumer preferences to better inform production and marketing strategies. We hypothesized that VOC concentration would increase as the radiation intensity increased. We also postulated that consumers would prefer basil with higher VOC concentrations and a more intense flavor.

## Materials and Methods

### Experimental Design Overview

This experiment was arranged in a split-plot design with two carbon dioxide (CO_2_) concentrations (two growth chambers) as the main factor and four radiation intensities as the subfactor, creating eight total treatments. Two hundred seedlings were grown per treatment with five plants per treatment randomly harvested for gas chromatography–mass spectrometry (GCMS) analysis. Each sample analyzed by GCMS contained only two leaves of one plant. The experiment was completed twice in time for GCMS analysis (reps 1 and 2).

For consumer sensory analysis, 600 plants were grown per treatment with four to eight leaves per sample administered to each participant. Ninety consumers participated in the first sensory analysis experiment (rep 2) sampling basil grown under four radiation intensity treatments (four total samples). Ninety-eight consumers sampled basil grown under two radiation intensities and two CO_2_ concentrations (four total samples) in the second sensory analysis experiment (rep 3). More in-depth explanations of the treatments and experimental conditions are described below.

### Seedling Production

Sweet basil “Nufar” (Johnny’s Selected Seeds, Fairfield, ME) was selected based on yield data from Walters and Currey (2015). Seeds were sown two per cell in stone wool cubes (2.5 × 2.5 × 4 cm, AO plug; Grodan, Roermond, Netherlands), with 200-cell flats placed in one of two walk-in growth chambers (Hotpack environmental room UWP 2614-3; SP Scientific, Warminster, PA) where treatments began immediately. Seeds and seedlings were irrigated overhead daily with deionized water supplemented with 12N–1.76P–13.44K water-soluble fertilizer providing (in mg ⋅ L^–1^) 100 nitrogen, 15 phosphorus, 112 potassium, 58 calcium, 17 magnesium, 2 sulfur, 1.4 iron, 0.5 zinc, 0.4 copper and manganese, and 0.1 boron and molybdenum (RO Hydro FeED; JR Peters, Inc., Allentown, PA) and magnesium sulfate (MgSO_4_) providing (in mg ⋅ L^–1^) 15 magnesium and 20 sulfur. Seedlings were thinned to one seedling per cell approximately 1 week after sowing. The air temperature setpoint was 23°C; air temperature was measured by a resistance temperature detector (Platinum RTD RBBJL-GW05A-00-M 36B; SensorTec, Inc., Fort Wayne, IN) every 5 s and logged by a C6 controller (Environmental Growth Chambers, Chagrin Falls, OH) with values reported in Table 1. Substrate temperature was measured with a thermistor (ST-100; Apogee Instruments, Logan, UT); leaf temperature was measured with an infrared thermocouple (OS36-01-T-80F; Omega Engineering, Inc., Norwalk, CT), and photosynthetic photon flux density (PPFD) was monitored with a quantum sensor (LI-190R Quantum Sensor; LI-COR Biosciences, Lincoln, NE) every 15 s with means logged every hour by a CR-1000 datalogger (Campbell Scientific, Logan, UT). Target CO_2_ concentrations of 500 and 1,000 μmol ⋅ mol^–1^ were maintained by injecting compressed CO_2_ to increase concentrations and scrub CO_2_ using a soda lime scrubber (Environmental Growth Chambers) to decrease concentrations. Concentrations were measured with a CO_2_ sensor (GM86P; Vaisala, Helsinki, Finland) and logged by a C6 Controller (Environmental Growth Chambers) every 5 s (Table 1).

**TABLE 1:** Target radiation intensity, actual radiation intensity, and average daily air, canopy, and substrate temperatures (mean ± SD) during the seedling growth stage (2 weeks).

	**Chamber**	**Radiation intensity (μmol ⋅ m^–2^ ⋅ s^–1^)**		**Temperature (°C)**		
		**Target**	**Actual**	**Air**	**Canopy**	**Substrate**
Rep 1	1	100	1020	23.00.0	24.40.9	21.90.7
		200	1910		26.41.0	−^*z*^
		400	4291		27.41.7	24.11.3
		600	5772		28.71.7	25.21.9
	2	100	944	22.91.8	24.50.9	22.21.0
		200	1882		24.01.1	22.00.9
		400	4321		26.91.5	23.71.4
		600	6152		27.72.1	24.61.9
Rep 2	1	100	992	23.00.4	24.20.5	21.70.6
		200	1843		26.50.9	−
		400	3846		27.41.6	24.31.5
		600	5559		29.32.0	25.31.9
	2	100	882	23.01.4	24.80.9	22.50.8
		200	1913		24.10.8	22.71.0
		400	3947		26.91.5	23.51.4
		600	58911		27.92.2	24.61.7

**^*z*^Data not collected.**

**FIGURE 1:**
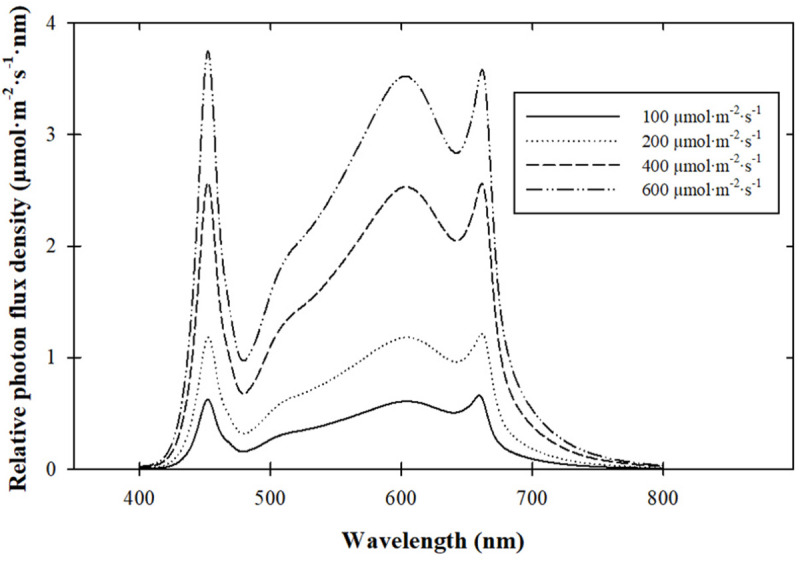
Spectral quality of light-emitting diode (LED) fixtures providing 20:40:40 blue:green:red radiation ratios (%), a red:far-red ratio of 13:1, and target radiation intensities of 100, 200, 400, or 600 μmol ⋅ m^– 2^ ⋅ s^–1^.

LEDs (Ray66 Indoor PhysioSpec; Fluence Bioengineering, Austin, TX) provided 20:40:40 blue:green:red radiation ratios (%), a red:far-red ratio of 13:1, and target PPFDs of 100, 200, 400, or 600 μmol ⋅ m^–2^ ⋅ s^–1^ for 16 h to create DLIs of 6, 12, 23, or 35 mol ⋅ m^–2^ ⋅ d^–1^, respectively (Figure 1). Fixture density and hanging height were adjusted to achieve target radiation intensities. Radiation intensity and spectrum were measured at four corners and in the center of the seedling flat with a spectroradiometer (PS-200; StellarNet, Inc., Tampa, FL) to quantify the intensities and spectrum (Figure 1) across the growing area with the variation reported in Table 1.

### VOC Data Collection and Analysis

Two weeks after sowing the two most recent, fully mature leaves of five plants per treatment, per replication (10 total plants), were detached, frozen, and stored at -20°C until GCMS analysis. In a method derived from Schilmiller et al. (2010), tissue was ground in liquid nitrogen, and an aliquot was placed in a 1.5 mL microcentrifuge tube containing 500 μL of methyl *tert*-butyl ether (MTBE) with 10 ng ⋅ μL^–1^ of tetradecane internal standard and gently rocked for 3 min. Samples were centrifuged to pelletize the tissue and 150 μL of supernatant was transferred to GS auto sampler vials. Samples were analyzed using an Agilent 7890A GC and single quadrupole MS with 5975C inert XL MS detector (Agilent, Santa Clara, CA). Standards were utilized to identify metabolites in addition to *m/z* values in the ChemStation database. Peak areas were integrated using MassLynx V4.1 QuanLynx software (Waters Corporation, Milford, MA). The compound concentrations were normalized to the sample tetradecane internal standard and leaf dry weight, then quantified using the standard calibration curves of 1,8 cineole, eugenol, linalool, and methyl chavicol with a tetradecane internal standard (Millipore Sigma; St. Louis, MO).

### Sensory Analysis

The protocol for the sensory analysis portion of this study was approved by the institutional review board of Michigan State University (MSU; East Lansing, MI, United States; STUDY000001369). The experimental procedure was thoroughly explained to all participants, and a written informed consent was obtained from each prior to participation.

Two consumer sensory panels were conducted using the Sensory Evaluation Laboratory in the Department of Food Science and Human Nutrition at MSU. The first compared four radiation intensity treatments, 100, 200, 400, and 600 μmol ⋅ m^–2^ ⋅ s^–1^ with plants grown in 500 μmol ⋅ mol^–1^ CO_2_. The second compared two CO_2_ concentrations, 500 and 1,000 μmol ⋅ mol^–1^, and two radiation intensities, 200 and 400 μmol ⋅ m^–2^ ⋅ s^–1^. Ninety and 98 participants, respectively, were recruited through the MSU Communication Arts and Sciences Paid Research Pool and screened prior to participation to ensure they had consumed basil in the past 4 months. Leaf samples were harvested 1–4 h prior to sampling to ensure freshness. Individual leaves were removed and rinsed in deionized water. Samples were dried with a salad spinner and/or through air drying. Depending on leaf size, four to eight leaves per sample were placed in cups.

Participants sat in a booth containing a roll-up pass-through door with a computer above the door, fluorescent lighting, and positive air pressure. Participants answered sensory evaluation and demographic questions through the Sensory Information Management System (SIMS 2000 version 6.0, Berkeley Heights, NJ) on the booth computer. Provided with water and saltine crackers, participants were served samples individually in a random order with three-digit blinding codes. Upon receipt of a sample, participants answered questions on a nine-point Likert scale ranging from *dislike extremely* to *like extremely* to describe how much they liked the sample over all, the aftertaste, appearance, aroma, flavor, texture, and leaf size; they rated the level of bitterness or sweetness; described what they liked and disliked about the sample; and shared any additional comments they had. Once a sample was evaluated, the sample was removed, and another sample was provided, prompting the same questions. Upon completion of the sensory evaluation, participants provided demographic information.

### Statistical Analysis

Analysis of variance was performed on VOC and Likert data using JMP (version 12.0.1; SAS Institute Inc., Cary, NC). Linear and quadratic regression analyses were conducted on VOC data using SigmaPlot (version 11.0; Systat Software Inc., San Jose, CA), and Tukey honestly significant difference test (*P* < 0.05) was conducted on Likert data using JMP. χ^2^-test (*P* < 0.05) was conducted to analyze word frequency using WordStat (version 8; Provalis Research, Montreal, Canada). Data were transformed to account for differences in values, variation, and sample size [(sample value - parameter average)/parameter SD], and then principal component analysis was conducted (using JMP). Biplots were created combining principal component analysis with loading plots. Factor analysis was conducted to determine the significant factors based on a rotated factor loading value of > 0.7. Correlations were determined by Pearson correlation coefficient at *P* < 0.05.

## Results

### Seedling Volatile Organic Compound Concentrations

**FIGURE 2:**
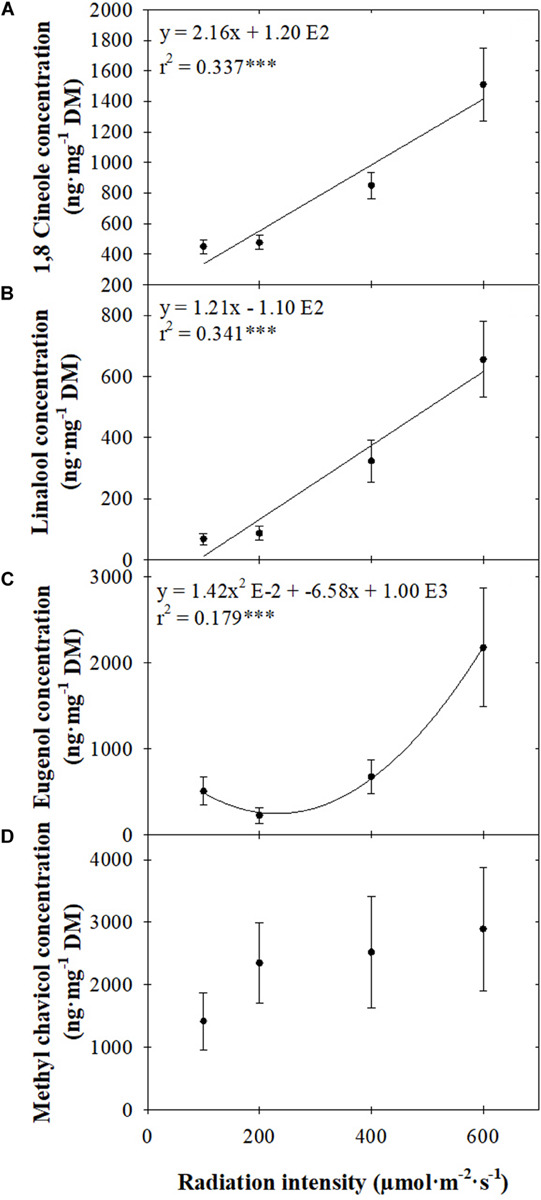
Concentrations [ng ⋅ mg^–1^ dry mass (DM)] of 1,8 cineole **(A)**, linalool **(B)**, eugenol **(C)**, and methyl chavicol **(D)** of sweet basil “Nufar” (*Ocimum basilicum*) seedlings grown under 100, 200, 400, or 600 μmol ⋅ m^–2^ ⋅ s^–1^ *photosynthetic* photon flux density (PPFD) for 2 weeks. Each symbol represents the mean of 20 plants ± SE. Lines represent linear or quadratic regression. ***Significant at *P* ≤ 0.001.

CO_2_ concentration did not influence VOC concentration (data not shown), so VOC data were pooled across both CO_2_ concentrations for each radiation intensity. The concentrations of both measured terpenoids increased linearly as radiation intensity increased, but the extent varied. Increasing the radiation intensity from 100 to 600 μmol ⋅ m^–2^ ⋅ s^–1^ increased 1,8 cineole concentration from 450 to 1,510 ng ⋅ mg^–1^ dry mass (DM; 2.4-fold), whereas linalool concentration increased from 67 to 655 ng ⋅ mg^–1^ DM (8.8-fold; Figures 2A,B).

The relationship between eugenol concentration and radiation intensity was quadratic. A 56% (283 ng ⋅ mg^–1^ DM) decrease in concentration occurred as radiation intensity increased from 100 to 200 μmol ⋅ m^–2^ ⋅ s^–1^, with an 8.7-fold (1,952 ng ⋅ mg^–1^ DM) increase as radiation intensity further increased from 200 to 600 μmol ⋅ m^–2^ ⋅ s^–1^ (Figure 2C). Average methyl chavicol concentration tended to increase as radiation intensity increased; however, because of large variations in concentration, differences between means were not significant (Figure 2D).

### Sensory Panel

The mean age of consumer panelists was 30.4 years, with an average of 1.2 adults and 0.4 minors in the household. Sixty-nine percent of panelists were female, 30% were male, 68% were Caucasian, and 19% were Asian. Average household income was $50,000, and 71% had at least a 4 year postsecondary education.

**FIGURE 3:**
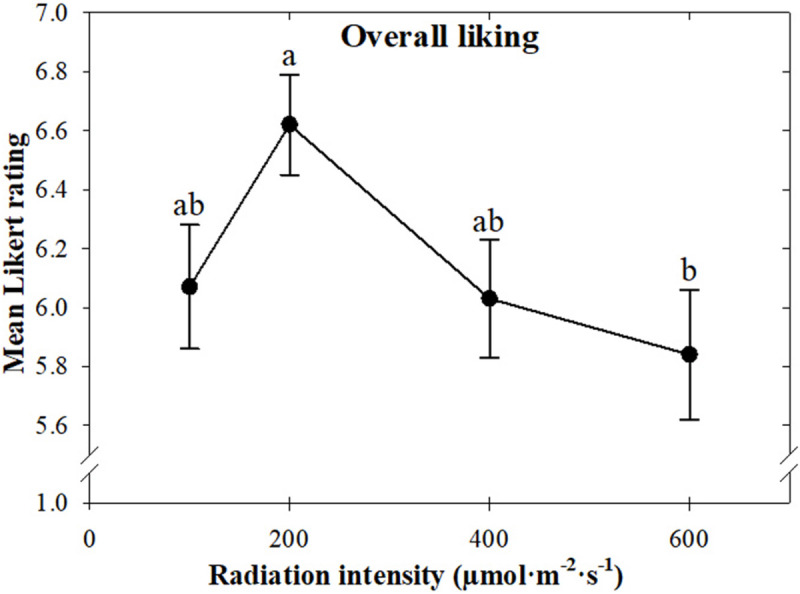
Mean overall liking of sweet basil “Nufar” (*Ocimum basilicum*) grown under 100, 200, 400, or 600 μmol ⋅ m^–2^ ⋅ s^–1^ photosynthetic photon flux density (PPFD), based on a nine-point Likert scale ranging from dislike extremely (1) to like extremely (9). Means not followed by the same letter are significantly different by Tukey honestly significant difference test (*P* < 0.05). Each symbol represents 90 responses ± SD.

**FIGURE 4:**
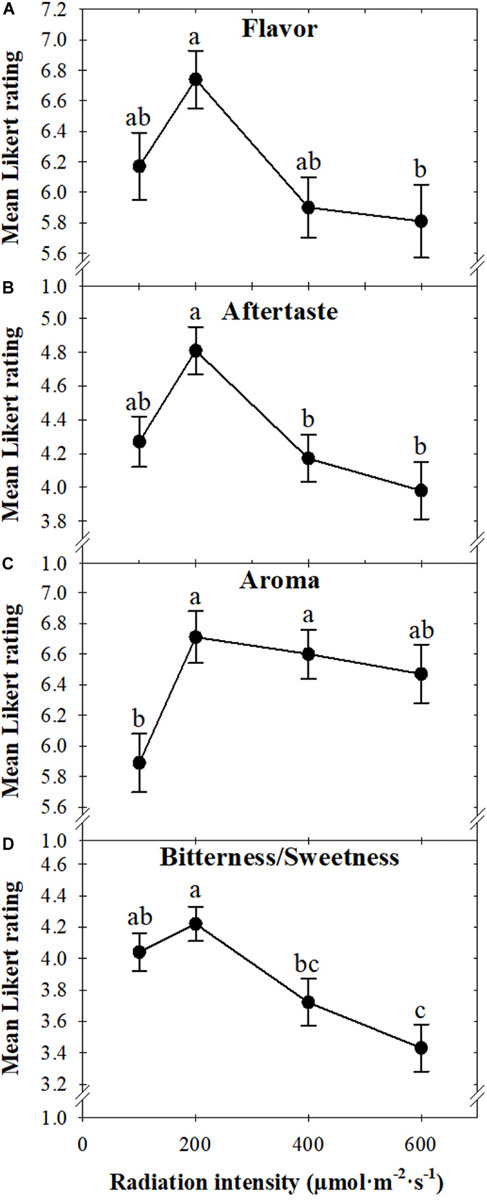
Mean flavor **(A)**, aftertaste **(B)**, and aroma **(C)** of sweet basil “Nufar” (*Ocimum basilicum*) grown under 100, 200, 400, or 600 μ*mol* ⋅ m^–2^ ⋅ s^–1^ *photosynthetic* photon flux density (PPFD), based on a nine-point Likert scale ranging from dislike extremely (1) to like extremely (9) and the bitterness/sweetness **(D)** based on a nine-point Likert scale ranging from extremely bitter (1) to extremely sweet (9). Means not followed by the same letter are significantly different by Tukey honestly significant difference test (*P* < 0.05). Each symbol represents 90 responses ± SD.

Overall, consumers preferred basil grown under a radiation intensity of 200 compared to 600 μmol ⋅ m^–2^ ⋅ s^–1^ (Figure 3). Flavor preference followed the same trend as overall preference, with consumers liking the flavor of basil grown under 200 more than 600 μmol ⋅ m^–2^ ⋅ s^–1^ (Figure 4A). Similarly, the aftertaste of plants grown under 200 μmol *⋅* m^–2^ ⋅ s^–1^ was preferred over those grown under 400 or 600 μmol ⋅ m^–2^ ⋅ s^–1^ (Figure 4B). Basil grown under 200 μmol *⋅* m^–2^ ⋅ s^–1^ was the least bitter. Increasing the radiation intensity to 400 or 600 μmol *⋅* m^–2^ ⋅ s^–1^ resulted in leaves that consumers rated as slightly bitter (Figure 4D). The aroma of plants grown under 100 μmol ⋅ m^–2^ ⋅ s^–1^ was the least preferred, with likability increasing as radiation intensity increased to 200 μmol ⋅ m^–2^ ⋅ s^–^*^1^* and then plateaued (Figure 4C).

**FIGURE 5:**
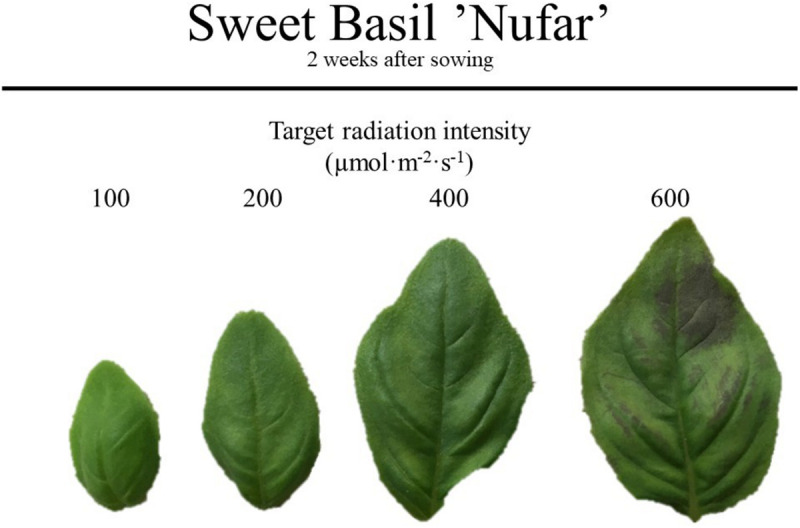
Leaves of basil used in the consumer sensory analysis panel. Sweet basil “Nufar” (*Ocimum basilicum*) was grown under radiation intensities of 100, 200, 400, or 600 μmol ⋅ m^–2^ ⋅ s^–1^ for a 16 h photoperiod to create daily light integrals of 6, 12, 23, or 35 mol ⋅ m^–2^ ⋅ d^–1^ for 2 weeks after sowing.

**FIGURE 6:**
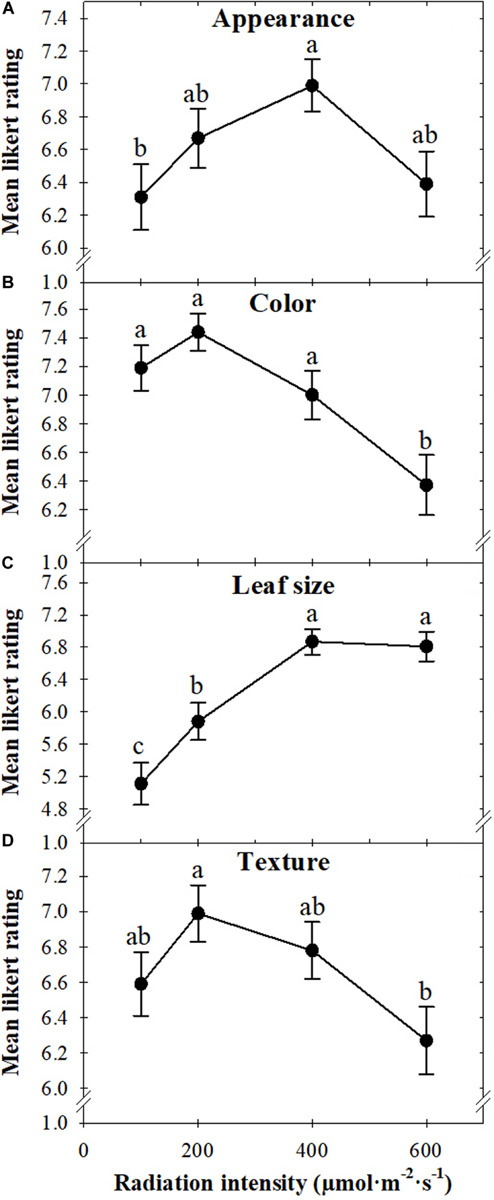
The mean appearance **(A)**, color **(B)**, leaf size **(C)**, and texture **(D)** of sweet basil “Nufar” (*Ocimum basilicum*) grown under 100, 200, 400, or 600 μmol ⋅ m^–2^ ⋅ s^–1^ photosynthetic photon flux density (PPFD), based on a nine-point Likert scale ranging from dislike extremely (1) to like extremely (9). Means not followed by the same letter are significantly different by Tukey honestly significant difference test (*P* < 0.05). Each symbol represents 90 responses ± SD.

Visually, the appearance of basil grown under 400 μmol ⋅ m^–2^ ⋅ s^–1^ was preferred to those grown under 100 μmol *⋅* m^–2^ ⋅ s^–1^ (Figures 5, 6A). The color of basil grown under 100, 200, or 400 μmol ⋅ m^–2^ ⋅ s^–1^ was preferred more than the color of plants grown under 600 μmol ⋅ m^–2^ ⋅ s^–1^ (Figures 5, 6B). The size of leaves produced under higher radiation intensities (400 or 600 μmol ⋅ m^–2^ ⋅ s^–1^) was preferred, with likability decreasing as radiation intensity decreased (Figures 5, 6C). Leaf texture preference followed a similar trend to overall liking in that panelists preferred plants grown under 200 μmol ⋅ m^–2^ ⋅ s^–1^ more than those grown under 600 μmol ⋅ m^–2^ ⋅ s^–1^ (Figure 6D).

**FIGURE 7:**
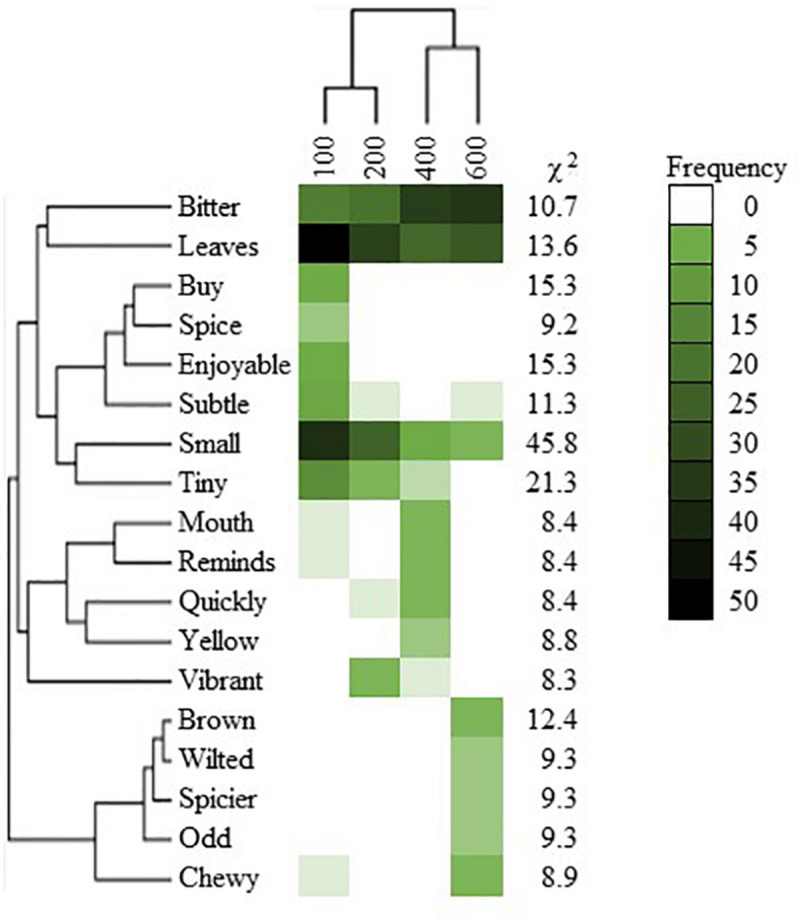
Frequency heat map of words used to describe sweet basil “Nufar” (*Ocimum basilicum*) grown under radiation intensities of 100, 200, 400, or 600 μmol ⋅ m^–2^ ⋅ s^–1^ photosynthetic photon flux density (PPFD). χ^2^ describes the goodness of fit; phylogenetic trees depict the cluster analysis relationship between words used and the relationship between words used and samples. All words reported exhibited differences *P* < 0.05 based on 90 respondents.

Based on word frequency, “bitter” was associated more with basil grown under higher radiation intensities (400 or 600 μmol *⋅* m^–2^ ⋅ s^–1^; Figure 7). The words “brown,” “chewy,” “odd,” “spicier,” and “wilted” were most strongly associated with plants grown under 600 μmol ⋅ m^–2^ ⋅ s^–1^ that, after rinsed for panel analysis, exhibited symptoms of leaf damage (Figure 5.). The words “mouth,” “quickly,” “reminds,” and “yellow” were most frequently used to comment on the 400 μmol ⋅ m^–2^ ⋅ s^–1^ grown plants. In comments regarding 100 and 200 μmol ⋅ m^–2^ ⋅ s^–1^ grown plants, “small” was frequently used; 100 μmol ⋅ m^–2^ ⋅ s^–1^ grown plants were also described with the words “buy,” “enjoyable,” “spice,” “subtle,” and “tiny,” and 200 μmol ⋅ m^–2^ ⋅ s^–1^ grown plants were described as “vibrant”.

In the second sensory panel, there were no differences in consumer preference due to CO_2_ concentration during production (data not shown). However, they confirmed the differences noted by the first sensory panel between basil grown under 200 or 400 μmol ⋅ m^–2^ ⋅ s^–1^. For example, basil grown under 200 μmol ⋅ m^–2^ ⋅ s^–1^ was less bitter than basil grown under 400 μmol ⋅ m^–2^ ⋅ s^–1^ (data not shown).

### Comparing Concentrations to Sensory Panel

**FIGURE 8:**
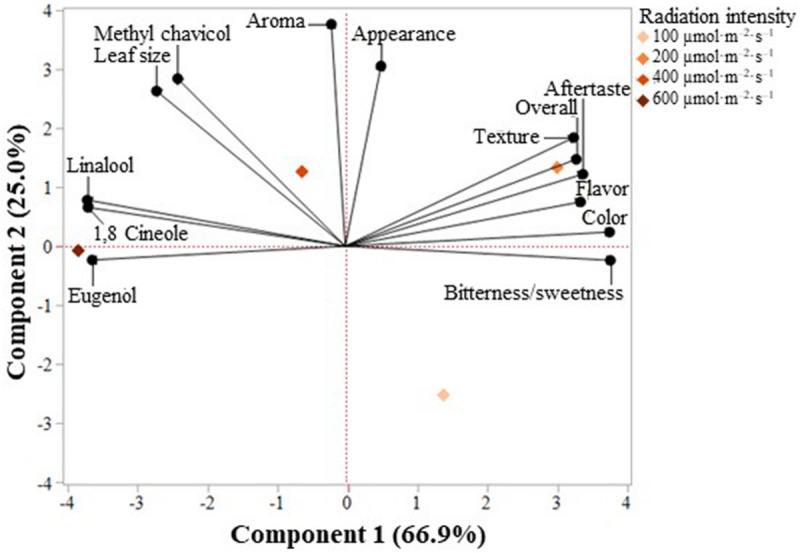
Principal component analysis (PCA) showing the biplot differentiation of sweet basil “Nufar” (*Ocimum basilicum*) grown under 100, 200, 400, or 600 μmol ⋅ m^–2^ ⋅ s^–1^ photosynthetic photon flux density (PPFD), based on consumer sensory preferences (*n* = 90) and the concentration of two terpenoids and two phenylpropanoids (*n* = 20).

A principal component analysis comparison of VOCs and consumer sensory preferences, represented by a biplot including basil samples grown under 100, 200, 400, or 600 μmol ⋅ m^–2^ ⋅ s^–1^, determined that two components accounted for 92% of the total data variability (Figure 8). Component 1 separated basil grown under 200 μmol ⋅ m^–2^ ⋅ s^–1^ from those grown under 600 μmol ⋅ m^–2^ ⋅ s^–1^, accounting for 66.9% of total data variability. The positive discriminating factors for component 1, associated with basil grown under 200 μmol ⋅ m^–2^ ⋅ s^–1^, were preference for aftertaste, bitterness/sweetness, color, flavor, overall liking, and texture, whereas the negative factors, greater 1,8 cineole, eugenol, and linalool concentrations, were associated with basil grown under 600 μmol *⋅* m^–2^ ⋅ s^–1^. Component 2, accounting for 25.0% of total data variability, separated basil grown under 100 μmol ⋅ m^–2^ ⋅ s^–1^ from those grown under 200, 400, and 600 μmol ⋅ m^–2^ ⋅ s^–1^. The positive discriminating factors were aroma, appearance, and leaf size preference and greater methyl chavicol concentration, associated with basil under 200, 400, and 600 μmol ⋅ m^–2^ ⋅ s^–1^. There are no negative discriminating factors.

**FIGURE 9:**
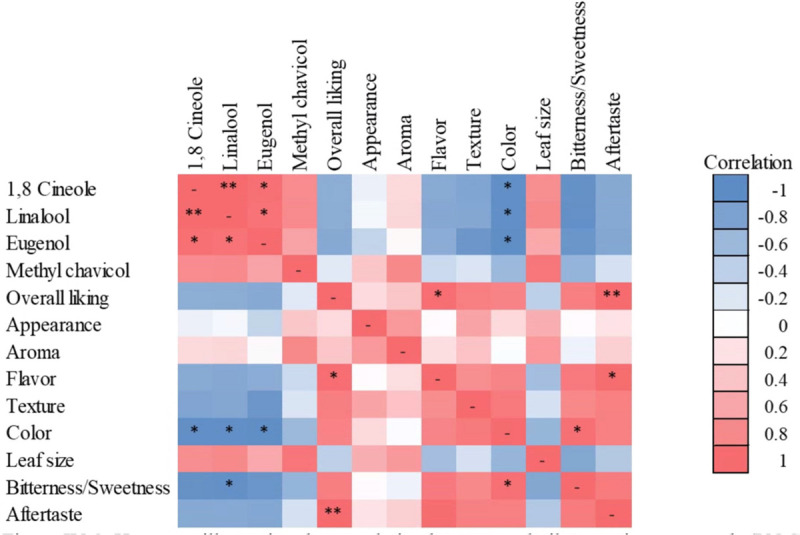
Heat map illustrating the correlation between volatile organic compounds (VOCs) and sensory preference characteristics of sweet basil “Nufar” (*Ocimum basilicum*) grown under radiation intensities of 100, 200, 400, or 600 μmol ⋅ m^–2^ ⋅ s^–1^ photosynthetic photon flux density (PPFD). Blue and red represent negative and positive correlations, respectively. Asterisks indicate significant correlations based on Pearson correlation *P < 0.05; **P < 0.01.

While 1,8 cineole, linalool, and eugenol concentrations were positively correlated with each other, they were negatively correlated with color, and linalool was negatively correlated with bitterness/sweetness (Figure 9). Additionally, aftertaste, flavor, and overall liking were positively correlated (Figure 9).

## Discussion

Sweet basil is consumed primarily for its distinct flavor. However, that flavor is highly variable based on cultivar genetics and the growing environment. An advantage to CEA production is that the growing environment can be monitored and adjusted to produce a consistent and potentially high-quality crop year-round. A major factor contributing to quality is the concentration of VOCs that contribute to crop flavor.

### Terpenoids

The linear increase in linalool and 1,8 cineole concentrations is congruent with previous research. Similar to Chang et al. (2008), who reported a ∼4-fold increase in linalool concentration as DLI increased from 5 to 25 mol ⋅ m^–2^ ⋅ d^–1^, we also found a nearly 4-fold increase as DLI increased from 6 mol ⋅ m^–2^ ⋅ d^–1^ (radiation intensity of 100 μmol ⋅ m^–2^ ⋅ s^–1^) to 23 mol ⋅ m^–2^ ⋅ d^–1^ (400 μmol ⋅ m^–2^ ⋅ s^–1^). This linear increase was likely due to increasing radiation intensity leading to increasing substrate availability (Chang et al., 2008). Although substrate availability may be the main limiting factor in monoterpenoid biosynthesis, other regulatory enzymes may also play a role (Muñoz-Bertomeu et al., 2006). For example, the level of 1-deoxy-D-xyluloase 5-phosphate synthase is directly positively correlated to the concentration of terpenoid and terpenoid products and can increase 2- to 9-fold in the presence of light (Estévez et al., 2000; Enfissi et al., 2005; Kim et al., 2005; Gong et al., 2006). Additionally, terpenoid concentration has been shown to correlate directly with terpene synthase activity (Iijima et al., 2004).

### Phenylpropanoids

In general, phenylpropanoid (eugenol and methyl chavicol) concentration increased as radiation intensity increased. However, as radiation intensity increased from 6 to 12 mol ⋅ m^–2^ ⋅ d^–1^ (100–200 μmol ⋅ m^–2^ ⋅ s^–1^), eugenol concentration decreased by 56%, whereas Chang et al. (2008) reported an increase in relative eugenol content as the DLI increased from 5 to 11 mol ⋅ m^–2^ ⋅ d^–1^ and a reduction from 11 to 14 mol ⋅ m^–2^ ⋅ d^–1^. Although the dip in relative eugenol content as radiation intensity increased occurred at different intensities (or DLIs), the dip still remains. However, similar to our results, Chang et al. (2008) reported that eugenol concentration increased ∼3-fold as DLI increased from 5 to 25 mol ⋅ m^–2^ ⋅ d^–1^; we found an over threefold increase when increasing the DLI from 6 to 23 mol ⋅ m^–2^ ⋅ d^–1^.

The concentration of phenylpropanoids is largely dependent on enzymatic concentration and activity, including PAL (Iijima et al., 2004). In a study comparing basil cultivar secondary metabolite variation, the cultivar with the highest phenylpropanoid concentrations (breeding line EMX) had PAL activity 2.8 times higher than the cultivar with the lowest phenylpropanoid concentration (“Sweet Dani”; Iijima et al., 2004). Also, the cultivar with phenylpropanoid concentration in between (breeding line SW) had intermediate PAL activity. They found several related sequences encoding PAL and determined PAL gene transcript levels mirrored phenylpropanoid concentration across the breeding lines or cultivars evaluated (Iijima et al., 2004). In basil, researchers have also reported increased levels of other compounds produced downstream of PAL in response to increased DLI including anthocyanins, total phenolics, and flavonoids (Dou et al., 2018).

Additionally, specific phenylpropanoids are also regulated further downstream. For example, the decrease in methyl chavicol concentration as leaves mature is caused partially by a reduction in chavicol *O*-methyltransferase and eugenol *O*-methyltransferase activity (Deschamps and Simon, 2006). Differential regulation may have led to the large variation in methyl chavicol concentrations in this seed-propagated cultivar.

### Compound Sensitivity

Preference or dislike for foods can be due to compounds present in very small concentrations (Dris and Jain, 2004). Human olfactory detection and recognition thresholds are the minimum concentrations of a compound that panelists can detect the presence of and recognize the compound, respectively (Patton and Josephson, 1957). Olfactory thresholds can serve as a basis for compound olfactory sensitivity. Thus, to account for differences in compound perception, odor activity values are calculated by dividing the compound concentration by the olfactory threshold. This allows for more accurate comparisons of compound contributions to overall olfactory perception. Although differences in detection thresholds in water, air, or other substrates and variations between studies and consumers make comparisons less precise, general trends can be drawn. Reported detection thresholds of 1,8 cineole, eugenol, linalool, and methyl chavicol in water are 1.1, 0.71, 0.087, and 6.0 μg ⋅ L^–1^, respectively (Czerny et al., 2008), whereas recognition thresholds are 4.6, 2.5, 0.17, and 16 μg ⋅ L^–1^, respectively (Zeller and Rychlik, 2006; Czerny et al., 2008), although linalool recognition threshold has also been reported as 5.0 μg ⋅ L^–1^ (Zeller and Rychlik, 2006). Therefore, in water, a higher methyl chavicol concentration is needed to be perceived as equally as 1,8 cineole, eugenol, or linalool. Both the detection and recognition thresholds for linalool are generally lower than 1,8 cineole, eugenol, and methyl chavicol; therefore, less linalool is needed to be perceived equally. In our experiment, linalool concentrations were lower than the other compounds measured, and methyl chavicol concentrations were higher; therefore, both compounds still had significant contributions to overall basil aroma.

In addition to variation in compound perceptibility, likability of compounds is an additional factor to consider in sensory analysis. To investigate the aroma acceptance of basil VOCs, researchers trained panelists to recognize linalool, 1,8 cineole, and eugenol by smelling progressively increasing concentrations of the standards (D’Antuono et al., 2007). Panelists then smelled diluted essential oil extracts from 24 basil cultivars and evaluated the perceived intensity of the compounds and overall acceptance. Although aroma acceptance varied greatly across cultivars, in general, high acceptance was not related to 1,8 cineole concentrations but correlated with high concentrations of linalool and low concentrations of eugenol. However, there were exceptions to this correlation, leading the authors to conclude that a balanced volatile concentration is needed for the greatest consumer acceptance (D’Antuono et al., 2007). Although the ratios between compounds in our study were not as varied as the D’Antuono et al., 2007 study because of the use of one cultivar, “Nufar,” differing ratios of compounds did occur. In our study, the ratio (%) of 1,8 cineole:eugenol:linalool:methyl chavicol concentration for plants grown under 100 μmol ⋅ m^–2^ ⋅ s^–1^ was 18:21:3:58, whereas plants growing under 600 μmol ⋅ m^–2^ ⋅ s^–1^ had a lower proportion of methyl chavicol with ratios of 21:30:9:40. However, the total concentration of 1,8 cineole, eugenol, linalool, and methyl chavicol was nearly threefold higher in plants grown under 600 μmol ⋅ m^–2^ ⋅ s^–1^ compared to those less than 100 μmol ⋅ m^–2^ ⋅ s^–1^. Therefore, this total increase probably played a larger role in consumer preference than the ratio of terpenoids and phenylpropanoids.

### Consumer Preferences

Consumer perception is influenced by many sensory modes including touch, sight, taste, and smell, where both taste and smell contribute to overall flavor. Although the exact number is unknown, researchers have estimated that consumers can generally distinguish 5,000 to 30,000 different odor qualities by smell, making their olfactory sense more diverse than the five basic tastes (Choi and Han, 2015). Although consumers can distinguish between many odors, they are more sensitive to flavor concentrations (Choi and Han, 2015). This was apparent in our data, as the aroma of basil grown under 100 μmol ⋅ m^–2^ ⋅ s^–1^ was the least preferred, whereas aroma likability was similar among plants grown under 200–600 μmol ⋅ m^–2^ ⋅ s^–1^. We postulate the reduced aroma preference of plants grown under 100 μmol ⋅ m^–2^ ⋅ s^–1^ was due to the low VOC concentration, thus weak aroma. While flavor and aftertaste preferences were greatest in plants grown under 200 μmol ⋅ m^–2^ ⋅ s^–1^, as VOC concentration increased with increasing radiation intensity, the flavor and aftertaste of plants grown under 600 μmol ⋅ m^–2^ ⋅ s^–1^ were not as well liked, suggesting a greater sensitivity to high VOC concentration contributions to flavor compared to aroma.

Bitterness is generally negatively correlated with consumer preference and is associated with harmful substances (Reineccius, 2005; Choi and Han, 2015). Therefore, the increased bitterness reported in basil grown under 400 and 600 μmol ⋅ m^–2^ ⋅ s^–1^ may have contributed to the flavor and aftertaste preferences.

Additionally, attributes such as texture and color contribute to overall consumer preference. Basil grown under 600 μmol ⋅ m^–2^ ⋅ s^–1^ exhibited the least-liked color and had among the lowest appearance and texture likability. The color was described by panelists as “brown” with symptoms of leaf damage due to the high radiation intensity. Additionally, consumers described the texture as “chewy” and “wilted.” This may be due to greater stomatal opening and gas exchange of plants grown under higher irradiances, which could have increased water loss and desiccation in the time between harvest and panelist evaluation (Davies and Kozlowski, 1975).

### Comparing Compound Concentrations and Preferences

In addition to component 1 of the principal component analysis separating basil grown under 600 from 200 μmol ⋅ m^–2^ ⋅ s^–1^, basil grown under 600 μmol ⋅ m^–2^ ⋅ s^–1^ was correlated with 1,8 cineole, eugenol, and linalool concentrations. Indeed, our GCMS analysis determined that basil grown under 600 μmol ⋅ m^–2^ ⋅ s^–1^ had the highest 1,8 cineole, eugenol, and linalool concentrations. However, contrary to our hypothesis that consumers would prefer basil with higher VOC concentrations, these higher concentrations were negatively correlated with sensory preference characteristics including bitterness/sweetness and color.

Basil grown under 200 μmol ⋅ m^–2^ ⋅ s^–1^ was correlated with consumer preferences for aftertaste, bitterness/sweetness, color, flavor, overall liking, and texture. This corresponds to the consumer sensory preference values discussed previously, where 200 μmol ⋅ m^–2^ ⋅ s^–1^ grown basil had among the highest Likert preference values for aftertaste, bitterness/sweetness, color, flavor, overall liking, and texture preference.

## Conclusion

Overall, increasing radiation intensity during indoor controlled-environment basil production increased terpenoid (1,8 cineole and linalool) and phenylpropanoid (eugenol) concentrations, whereas CO_2_ concentration had no effect. While overall liking was correlated with aftertaste and flavor preference, contrary to our hypothesis, increasing VOC concentrations to increase flavor did not improve flavor preference. Many consumer sensory preference characteristics (aftertaste, bitterness/sweetness, color, flavor, overall liking, and texture) were correlated with basil grown under a radiation intensity of 200 μmol ⋅ m^–2^ ⋅ s^–1^, which had among the highest consumer preference values. This leads us to conclude that consumers prefer to detect the characteristic basil flavor made up of 1,8 cineole, eugenol, and linalool, which is not as prevalent in basil grown under 100 μmol ⋅ m^–2^ ⋅ s^–1^, but too high of VOC concentrations when grown under 400 and 600 μmol ⋅ m^–2^ ⋅ s^–1^ lead to a lower consumer preference.

## Data Availability Statement

The raw data supporting the conclusions of this article will be made available by the authors, without undue reservation, to any qualified researcher.

## Ethics Statement

The studies involving human participants were reviewed and approved by the Michigan State University IRB. The patients/participants provided their written informed consent to participate in this study.

## Author Contributions

KW, RL, and BB conceptualized and designed the study, KW performed the experiments, conducted data analysis, and prepared the manuscript. RL and BB obtained funding and revised the manuscript. All authors contributed to the article and approved the submitted version.

## Conflict of Interest

The authors declare that the research was conducted in the absence of any commercial or financial relationships that could be construed as a potential conflict of interest.
